# Rehabilitation of a Patient after a Transtibial Amputation: A Case Report

**DOI:** 10.7759/cureus.30773

**Published:** 2022-10-27

**Authors:** Sayali S Mahulkar, Priyanka A Telang, Sakshi P Arora

**Affiliations:** 1 Physiotherapy, Ravi Nair Physiotherapy College, Datta Meghe Institute of Medical Sciences, Wardha, IND; 2 Community Health Physiotherapy, Ravi Nair Physiotherapy College, Datta Meghe Institute of Medical Sciences, Wardha, IND

**Keywords:** artificial limb, physiotherapy, rehabilitation, preprosthesis, transtibial amputation

## Abstract

Amputation is the surgical removal of a body part or limb caused by excessive tightness, pathological conditions, or a surgical procedure on an extremity. Transtibial amputation involves removing the feet, ankle, distal part of the tibia, and fibula with the surrounding structures like connective tissues and other structures surrounding the distal part of the bone. This type of surgery has a high rate of contraindications but has adequate indications; it maintains a therapeutic technique with a high clinical price and, in general, lifesaving value. The primary objectives of rehabilitation are to improve normal and affected extremity power, patient mobility, aerobic capacity, coordination and balance, and independence in performing activities of daily living. In this case, the patient was a 50-year-old man who complained of pain in his left lower limb and had an ulcer on his left foot. Initially, it was small in size when noticed by the patient but gradually progressed to a large wound of 7x6cm over the medial side, with foul-smelling discharge, associated with blackish discoloration. Walking aggravated foot pain, which was relieved by standing still, medications, and rest. On investigation, the patient was diagnosed with gangrene in the left lower limb and referred for rehabilitation to the physiotherapy department after surgery. This case study provides information on the recovery of a patient with the help of prosthetic devices.

## Introduction

Lower extremity amputations may be classified as one of the major events that can change a person's life and functional abilities. A lower extremity amputation can be categorized in two ways: above-the-knee amputations (AKA) or below-the-knee amputations (BKA). Following surgery [1], below-the-knee amputations provide the person with more balance, stability, strength, and mobility following surgery [1], whereas an above-the-knee amputation makes recovery and return to normal functioning much more difficult. A below-knee amputation could be a transtibial amputation that requires the separation of the foot, mortise joint, distal shinbone, and bone in the lower limbs with connected structures of soft tissues (BKA). There are more than 3500 amputations performed due to trauma. Trauma, congenital deformities, and diabetic neuropathy are some of the possible causes of lower extremity amputation. Traditional approaches remain important. Nonetheless, it is reasonable to state that both virtual reality and software-based rehabilitation programs are progressively being created and receiving increasing support [2]. Because of the serious adverse effects of AKA, BKA is preferred. Lower limb amputation rates have been dropping in recent years, but it should remain a therapeutic option of major clinical and often life-saving value if adequate advice is provided. Even in developed nations, lower limb amputation (LLA) remains a health concern that needs rehabilitation and long-term care [3].

Generally, below-knee amputation is preferred when there are complications like wet gangrene or non-healing ulcers. The fundamentals of amputee rehabilitation are reviewed, including pre-amputation to rehabilitation into the job force and community. The successful use of prostheses following lower-limb amputation (LLA) is dependent on both physical and psychological rehabilitation [4]. Treatment of post-amputation pain disorders, including phantom limb pain, can be difficult, but there are innovative pain-control methods. Long-term care of both the remaining and opposite limbs is critical to reducing the risk of amputations and improving the long-term quality of life and function [5]. Amputation cannot be prevented by conservative or surgical techniques. This case report describes exercise routines, training programs, and environmental changes that have been proven to be beneficial in amputee rehabilitation. Flexibility, muscle strength, aerobic conditioning, and balance and gait are the four key components of the exercise routines given here. The program, which is overseen and directed by a physician, includes interventions by physical, occupational, and recreational therapists, as well as environmental changes that have been shown to aid in amputee recovery.

## Case presentation

A 50-year-old male was referred to the physiotherapy department for pre-prosthetic rehabilitation and prosthetic prescription after transtibial amputation (TTA). He had complained of an ulcer on his left foot, which was non-healing in nature, for which he visited the hospital. A TTA was done on his left leg as a lifesaving procedure after severe infection and gangrene. He also suffered initially from uncontrolled diabetes and hypertension. He had been a chronic alcoholic for 20 years. The differential diagnosis of the patient was varicose veins, peripheral nerve injury, deep vein thrombosis, infections, and cellulitis.

Operative notes 

The patient underwent intra-arterial thrombolysis of the left lower limb. The lesion was crossed with a Terumo Guide Wire cross-over sheath followed by an angiogram and selective catheterization of the anterior tibial artery. The catheter was placed in the thrombotic arterial segment.

Furthermore, the patient presented with severe wasting malnutrition due to increasing weight loss, type 2 diabetes, and depression. During surgery, mechanical wound suction was used. The patient was advised to return to the hospital for a check-up to assess his recovery, wound changes, and wound healing time.

Clinical findings

The patient had complaints of discomfort and the growth of an ulcer over the left foot, which had not healed six months before presentation. Edema developed and the skin on the left lower foot turned dark. A discharge from the foot was noticed, which smelled foul. After all the investigations were done, the patient was diagnosed with left lower leg gangrene. On July 30, 2022, the patient underwent surgery in which transtibial amputation was performed.

According to observations, the patient's body type was mesomorph. The goniometer determined that the range of motion was within normal limits, as indicated in Table 1. The results of the Manual Muscle Testing (MMT) are shown in Table 2. The Barthel Index was used to measure activities of daily life, and the patient received a score of 7. This instrument determines the degree of dependence while performing activities of daily living.

**Table 1:** Range of motion: Before and after the rehabilitation ROM: Range of motion

Movement	Pre-rehab ROM: Right side	Pre-rehab ROM: Left side	Post-rehab ROM: Right side	Post-rehab ROM: Left side
Hip flexion	0-100	0-50	0-120	0-60
Hip extension	0-20	0	0-30	0-10
Hip abduction	0-40	0-20	0-50	0-30
Hip adduction	0-20	0-10	0-30	0-20
Knee flexion	0-120	0	0-120	0-5
Knee extension	0	0	0	0
Ankle plantarflexion	0-30		0-50	-
Ankle dorsiflexion	0-15		0-15	-

**Table 2:** Manual Muscle Testing: Before and after rehabilitation

Muscles	Pre-rehab	Post-rehab
Knee extensor	2	3
Knee flexors	3	4
Hip extensors	3	4
Hip flexors	2	3
Gluteus medius	3	4
Gluteus maximus	3	4

Pain assessment

A pre-rehabilitation assessment was conducted on August 14, 2022, using the Visual Analogue Scale. It was noted at 8/10 at rest and 9/10 on slight movement. Another assessment was conducted after the completion of rehabilitation. The Visual Analogue Scale was used for this and it noted 3/10 at rest and 4/10 on slight movement. This indicated that after rehabilitation, the pain had subsided.

A neurological examination was done on both the affected as well as unaffected limbs. Myotomes and dermatomes were tested, and no changes were noted in sensations, and all the spinal nerve roots were normal. Deep tendon reflexes (DTR) were fully functional.

As shown in Table 1, the range of motion of the left side improved post-rehabilitation. As indicated in Table 2, post-rehabilitation muscle strength also increased when compared to the pre-rehabilitation stage.

Diagnosis

Color Doppler analysis of the left lower limb arteries revealed triphasic flow, reduced flow in the left lower limb artery, common iliac artery (CIA), external iliac artery (EIA), internal iliac artery (IIA), superficial femoral artery (SFA), popliteal artery (POP), anterior tibial artery (ATA), posterior tibial artery (PTA), and wall thickening in the arteries of the left leg.

A Computed Tomography scan of the brain indicated chronic ischemia of the small vessel alterations in both deep white matter and periventricular areas with widespread cerebral atrophy without contrast.

Timeline 

On July 25, 2022, a patient was admitted with a complaint of an ulcer over the lower limb and pain in the left lower limb. He was operated upon on August 13, 2022, and the very next day, on August 14, 2022, a physiotherapy referral was given.

Intervention 

Short-term postoperative goals were to reduce pain, increase lower limb movement, maintain function in the remaining leg and stump, maintain peripheral circulation, and maintain respiratory function. The long-term goals were to maintain balance, undergo prosthetic training, and prevent contractures.

Table 3 showcases the rehabilitative interventions planned for the patient from postoperative day 1 to week five. It includes information on a home exercise program, notes on follow-up, and results.

**Table 3:** The patient's postoperative rehabilitation plan POD: Postoperative day

Time	Therapeutic intervention
POD 1 to Week 1	To maintain airway clearance, deep breathing exercises were prescribed. To maintain range of motion, active range of motion of the unaffected lower limb, active range of motion of the upper limb, and resistance exercises like isometrics of the quadriceps and gluteal muscles were prescribed. Passive range of motion (extension) of a transtibial residual limb to be improved.
Week 2– Week 3	Mobility exercises in bed, static quadriceps and hamstrings, and bedside sitting. Ankle toe movements of the right side, dynamic quadriceps, shoulder scapular set, and ambulation with a a walker. Pneumatic compression was given to the patient to relieve pain and increase arterial blood flow in the distal limbs.
Week 3 – Week 4	The pre-prosthetic phase of the amputee's lower limb was started. Icing, ultrasound, massage, and biofeedback techniques are used to treat phantom limb pain. Edema control measures include bandaging, rigid dressing, and a pneumatic post-amputation mobility aid. Stump socks were used to take care of the stumps. Proper residual limb positioning was given.
Week 4 - Week 5	Walking aids were taught to the individual in order to prevent falls, and ambulatory education with a walker began. Gait training using a parallel bar was performed (Figure 1). The exercise was of the proprioceptive variety. Initially, ambulation was limited to 100m with the help of a walker, then progressed to the corridor. The distance of the walk was gradually increased and later moved to stair climbing with the help of crutches.
Home exercise program	The patient was recommended to continue physiotherapy after discharge from the hospital. Detailed instructions were provided to undertake all activities and other duties at home with specified repetitions and weights. The patient displayed an improvement in hip joint movement, knee movements of the amputee limb, and a decrease in discomfort after physiotherapy treatment.
Follow-up and outcomes	The patient was able to perform most daily activities and had no pain in his amputated limb. The knee of the amputated leg had an increased range of motion. The patient was eager to participate in physiotherapy and was highly motivated. The patient was also given information on home workout regimens and was urged to modify his posture. There was an improvement in the range of motion of the hips. As the physiotherapy treatment progressed, there was a marked improvement in the patient's condition.

**Figure 1:**
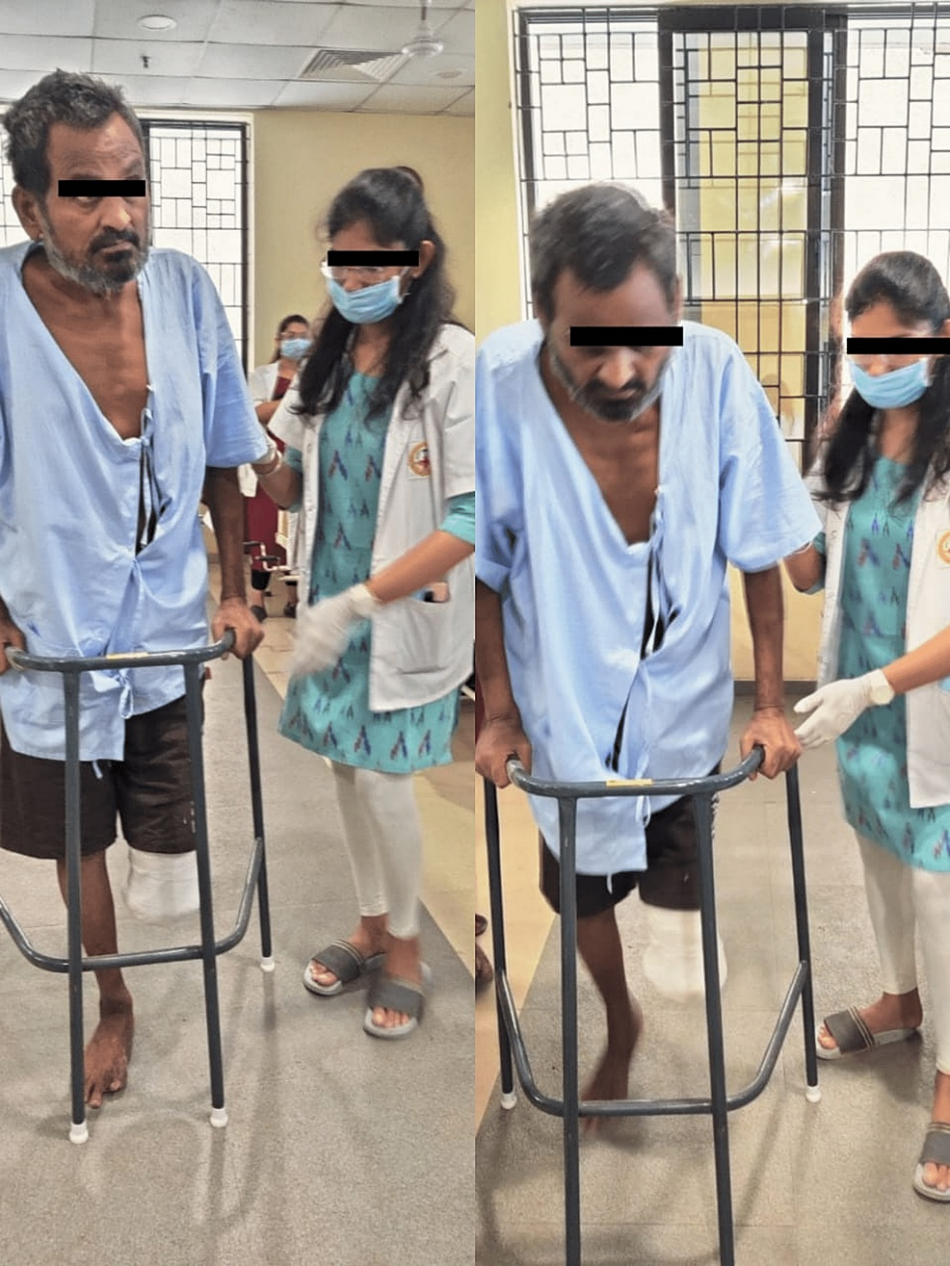
The patient undergoing weight bearing and gait training

## Discussion

Below-the-knee amputation involves removing the foot, ankle joint, and distal tibia and fibula with related soft tissue structures. There are four major categories of indications for proceeding with a below-the-knee amputation. These include urgent cases where severe infections are spread, trauma or injury, tumors, or poor blood flow that cannot be fixed.

Strengthening of the lower limb muscles, gait training, exercises to maintain balance, and training to make patients independent were the important aims of physical therapy during the phase of recovery. Supervised walking, muscle strengthening, balance exercises, gait training, and functional training programs demonstrated small-to-large effect-size gait performance improvements in people with lower limb amputation. Self-selected gait speed was the most consistent outcome measure. Exercise programs emphasizing resisted gait and functional training were more effective than supervised walking [6]. Gait analysis combined with sound clinical judgment plays an important role in elucidating the factors involved in pathologic prosthetic gait and the selection and effects of available interventions to optimize it [7].

Mirror therapy, motor imagery, and virtual visual feedback, according to Laura Herrador Colmenero et al., reduce phantom limb pain; however, there is limited scientific evidence supporting their effectiveness [8]. Physical medical assistance during and after transtibial amputation results in a satisfactory recovery. This report demonstrates that physical treatment is beneficial during the pre-amputation, after the amputation, and during prosthetic rehabilitation.

## Conclusions

Physical therapy exercises help in maintaining the quality of life of patients. Exercises like strengthening, postural flexibility, and dynamic equilibrium exercises help the patient recover and help them do activities of daily living, and make the patient independent. Strengthening exercises help to strengthen the muscles of the amputated limb. Equilibrium exercises maintain balance. All these exercises help prepare the patients for the prosthesis fitting and help determine the suitability of the prosthetic device for walking. Early walking aids may be used. These rehabilitation protocols will help the patient recover well.
